# Comparison of Retinal Nerve Fiber Layer Thickness Measurements in Healthy Subjects Using Fourier and Time Domain Optical Coherence Tomography

**DOI:** 10.1155/2012/107053

**Published:** 2012-05-29

**Authors:** Isabel Pinilla, Elena Garcia-Martin, Miriam Idoipe, Eva Sancho, Isabel Fuertes

**Affiliations:** ^1^Ophthalmology Department, Lozano Blesa University Hospital, c/ San Juan Bosco 15, 50009 Zaragoza, Spain; ^2^Aragones Institute of Health Science, IIS Aragon, C/ Gómez Laguna 25, 50009 Zaragoza, Spain; ^3^Ophthalmology Department, Miguel Servet University Hospital, c/ Isabel la Católica 1-3, 50009 Zaragoza, Spain

## Abstract

*Purpose*. To compare the retinal nerve fiber layer (RNFL) measurements using two different ocular coherence tomography (OCT) devices: Cirrus Fourier domain OCT and Stratus time domain OCT. To analyze reproducibility of Fourier domain measurements in healthy subjects. *Methods*. One hundred and thirty-two eyes of 132 healthy subjects were scaned on the same day with both instruments, separated by 10 minutes from each other. Thickness of quadrant, average and the 12 different areas around the optic nerve were compared between Cirrus and Stratus. Repeatability, intraclass correlation coefficients (ICCs), and coefficients of variation (COVs) were calculated in RNFL measurements provided by Fourier domain device. *Results*. The average thickness in the optic cube was 95.50 **μ**m using Cirrus and 97.85 **μ**m using Stratus. Average thickness and temporal quadrant showed significant differences using Cirrus and Stratus methods. Reproducibility was better with Fourier domain OCT (mean COV of 4.54%) than with Stratus time-domain OCT (mean COV of 5.57%). *Conclusions*. Both scan options give reproducible RNFL thickness measurement, but there are differences between them. Measurements obtained using Fourier domain device show better reproducibility.

## 1. Introduction

Optic nerve diseases, including glaucoma and other neurological pathologies, need and objective, quantitative, and sensitive method to assess retinal nerve fiber layer (RNFL) thickness both for diagnosing and monitoring their progression. The importance of RNFL thickness determination in the early diagnosis of glaucoma cannot be overstated, because RNFL thinning may in theory be the earliest structural change clinically detectable and has been shown to precede functional loss by as much as 5 years [1]. RNFL thickness maps can also be potentially used for a thorough evaluation of the RNFL in the longitudinal monitoring optic nerve disease, which is extremely important in the clinical management of a life-long disease.

Optical coherence tomography (OCT) is nowadays an important diagnostic tool for retinal diseases in the clinical practice. It provides cross-sectional or three-dimensional images by measuring the echo time delay and magnitude of backscattered or back-reflected light. The OCT gives a kind of optical biopsy with quantitative and reproducible measurements of macular and RNFL thickness parameters using near-infrared light [2, 3]. OCT was first developed as a research tool in 1991 obtaining two-dimensional images [4, 5] using low-coherence interferometry to measure the time delay of back-scattered light from different layers of the retina. Time domain OCT (TDOCT) technology was the first introduced. The standard in OCT retinal imaging was the Stratus-OCT (Carl Zeiss Meditec Inc, Dublin, California, USA). Stratus OCT was widely used in clinical settings, and provided detailed cross-sectional images and quantitative information of the retina with an axial resolution of 10 *μ*m and a scan velocity of 400 axial scans per second [6, 7]. Stratus OCT has demonstrated its ability to detect RNFL loss [8]. For the past last years, improved OCT devices employing spectral domain OCT (SDOCT) or Fourier-domain OCT (FDOCT) have been introduced in the clinical practice. Fourier domain detection can measure all echoes of light from different delays simultaneously. This technology features greater scan acquisition speed, higher resolution images, and more reproducible measurements. The higher acquisition speed reduces the eye motion artefacts and enables a better delineation of the retinal layers [9]. It is then possible to detect and segment the retinal structures in each raster OCT image [10]. FDOCT provides a faster scanning of the tissue achieving an axial resolution up to five times higher and imaging speeds up to 60 times greater than conventional OCT [11–14]. Cirrus high definition (HD) OCT (Carl Zeiss Meditec) is a FDOCT that has an axial resolution of 5 *μ*m and a scan velocity of 27,000 axial scans per second. 

Several studies have reported the importance of RNFL thickness determination in the early diagnosis and managing of optic nerve conditions, such as glaucoma [15], band atrophy with or without chiasmal compression [16], demyelinating diseases [17, 18], and optic neuritis. This topic based on OCT can reveal changes in RNFL thickness before visual field defects appear [19]. It is important that these new techniques are capable of marking accurate, reliable, and reproducible measurements, because the results of the RNFL thickness evaluations may vary widely according to the devices used.

The repeatability and reliability of retinal thickness measurements using Stratus OCT has been probed in several studies [20, 21]. Different studies had also demonstrated that FDOCT are very reliable devices [18, 22, 23].

An essential quality in determining the utility of a device for clinical use is its measurement reproducibility. The goal of our present study was to compare the RNFL measurements in healthy persons using both OCT methods and to determine the reproducibility of RNFL measurements with both instruments.

## 2. Material and Methods

We carried out a prospective cross-sectional study including 132 consecutive healthy subjects who were imaging with Stratus OCT and Cirrus HD OCT on the same day. One randomly selected eye of each subject was analyzed. All the procedures were conducted in accordance with the principles of Helsinki Declaration and the experimental protocol was approved by the local Ethics Committee. Detailed consent forms were obtained from each subject.

For Cirrus HD and Stratus instruments the RNFL is presented on two circular charts, one with 12 equal sectors each representing one clock hour and the other with four equal 90° sectors, each representing one quadrant (temporal: 316–45°on unit circle, superior: 46–135°, nasal: 136–225° and inferior quadrant: 226–315°). The chart displays microns of the RNFL thickness. Average RNFL thickness (0°–359°) is also displayed.

All the subjects underwent a comprehensive ophthalmic examination including medical, ocular and family history, best corrected visual acuity (BCVA), visual field, slit lamp biomicroscopy, intraocular pressure (IOP) measurements with Goldmann applanation tonometry, and fundoscopy examination. 

The included persons had BCVA of 20/25 or better according to the Snellen scale, a normal visual field (VF) examination, and no history of ocular or neurological disease. Exclusion criteria were the presence of significant refractive errors (more than 5 diopters of spherical *equivalent refraction  *or 3 diopters of astigmatism), IOP of 21 mmHg or higher, media opacifications, systemic conditions that could affect the visual system, a history of ocular trauma or concomitant ocular diseases, including a previous history of retinal pathology, glaucoma, laser therapy, or ocular pathologies affecting the cornea, lens, retina, or optic nerve. 

The VF was assessed using a Humphrey Field Analyzer (Carl-Zeiss Meditec, Dublin, CA). A SITA Standard strategy (program 30-2) was used to decrease the duration of the exam.

Each subject was imaged with the two OCTs on the same day without dilating the pupil. Fast RNFL thickness (3.46) and optic disc cube 200 × 200 were the scan acquisition protocols used for measuring the peripapillary RNFL thickness with the TDOCT and Fourier-domain FDOCT, respectively. This diameter was set by Schuman et al. because it is large enough to avoid the overlap with the optic nerve head in almost all the eyes and yet allow measurement in an area with thicker RNFL [2].

In order to study the reproducibility of the measurement, all the subjects underwent 3 different 3.4 mm diameter circular scans centered on the optic disc with the Cirrus HD OCT, with 10 minutes of time between each other. To have our own Stratus OCT reproducibility, 90 of the total subjects had three different scans following the same procedure. The two OCTs were used in random order to prevent any effect of fatigue bias.

Between scan acquisitions, there was a time delay and subject position and focus were randomly disrupted, meaning that alignment parameters had to be newly adjusted at the start of each image acquisition. No manual correction was applied to the OCT output. An internal fixation target was used because it has previously been shown to give the highest reproducibility [2]. 

Following the recommended procedure for scan acquisition, the subject's pupil was first centered and focused in an iris viewing camera on the system data acquisition screen, and then the system's line-scanning ophthalmoscope was used to optimize the view of the retina. The OCT scan was aligned to the proper depth and patient fixation, and system polarization was optimized to maximize the OCT signal. 

Three repetitions of optic disc cube 200 × 200 scans in each eye were performed using both OCT devices. The Cirrus HD OCT optic disc protocol generates a map with average RNFL thickness, quadrant RNFL thickness (superior, nasal, inferior and temporal), and 12 clock hours of 30° RNFL thickness. The numeration of the hour sectors was assigned from position H1 to H12 in the clockwise direction for the right eye and in the counterclockwise direction for the left eye. Same data are provided by Stratus OCT. 

The presence of defects in the RNFL can be detected using both devices (Stratus and Cirrus HD OCT) and is provided by the comparison of measurements from each patient with the normative database of each instrument.

Two investigators (I. Pinilla, E. Garcia-Martin) judged the scans to be of acceptable quality and selected only the scans were optic disc was centered and focused within the circular scan, using fundus images captures on OCT scan printout. All the scans had continuous lines for demarcating the RNFL border. Only subjects with good images obtained with both OCTs were selected for the study. Cirrus HD and Stratus OCT determine the quality of images using the signal strength measurement that combines signal-to-noise ratio with the uniformity of the signal within a scan and is measured on a scale of 1 to 10, where 1 is categorized as poor image quality and 10 as excellent image quality. Only images with a score higher than 7 were evaluated in our study. Three series of good quality scans were obtained for each option. Twenty-nine images with artifacts, missing parts, or showing seemingly distorted anatomy were excluded [24]. To obtain good quality and centered images, ten eyes required repeat scan acquisition using the Cirrus HD OCT and twenty-three eyes using the Stratus OCT. 

### 2.1. Statistical Analysis

 Statistical software (SPSS 15.0, SPSS Inc., Chicago, IL) was used for statistical analysis. The Kolmogorov-Smirnov test was used to assess sample distribution. The Cirrus HD values were analyzed using the mean of the three measurements. The average RNFL thickness and the RNFL in each quadrant and in each 30° segment were tested for statically disparity between both OCT instruments. All the results were expressed as mean ± standard deviation (±SD). 

Differences between Cirrus HD and Stratus RNFL measurements in each group were also compared using a Student's *t*-test for paired data. Values of *P* < 0.05 were considered to be indicative of statistically significant differences. Comparisons of RNFL thickness between the two instruments were graphically represented. 

Variability was assessed by computing for each parameter the standard deviation of the mean, repeatability, coefficient of variance and intraclass correlation coefficient. For each parameter, the coefficient of variation (COV) was calculated as the standard deviation divided by the average of the measurement value and expressed as a percentage. Most authors consider that devices with a COV less than 10% have high reproducibility, while a COV less than 5% indicates very high reproducibility [22]. To assess the reliability of the repeated measurements, the intraclass correlation coefficients (ICCs) for absolute agreement were calculated. They measure the concordance for continuous variables and correct correlations for systematic bias. The ICC interpretation that we used was slight reliability (for values between 0 and 0.2), fair reliability (from 0.21 to 0.4), moderate reliability (values between 0.41 and 0.6), substantial reliability (values from 0.61 to 0.8), and almost perfect reliability (for values of intraclass correlation coefficients higher than 0.81). Bland and Altman plots were used to assess agreement. Reproducibility of both devices was also represented by displaying the differences between measurements by the two instruments against the mean of the two measurements (Bland-Altman plots) [25].

## 3. Results and Discussion

**Figure 1:**
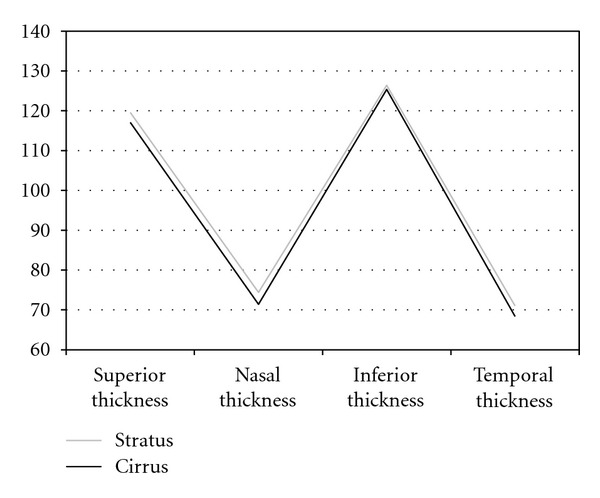
Representation for retinal nerve fiber layer thicknesses in the four quadrants using Cirrus HD and Stratus OCT devices. Measurements were higher with Cirrus OCT.

Table 1 gives the mean values and standard deviation of the RNFL in global thickness and in each parapapillary quadrant in all the subjects. Significant differences were found in the global thickness using both instruments; this value was 2.37 microns superior with Stratus than with Cirrus HD OCT (*P* = 0.003). Significant differences were only found between the temporal quadrants measurements with both instruments (2.21 *μ*m higher using Stratus; *P* = 0.046). When the different 12 segments around the optic disc were analyzed, we found significant differences in sectors H1, H4, H5, H8, H11, and H12 (see Table 1). The quadrant thickness differences between both OCT devices are shown in Figure 1. The graph demonstrates that measurements of RNFL thickness were thicker with the Stratus OCT than with the Cirrus HD OCT. Also, the inspection of the plots reveals discrepancy between RNFL thickness measurements obtained by both OCT instruments.

**Figure 2:**
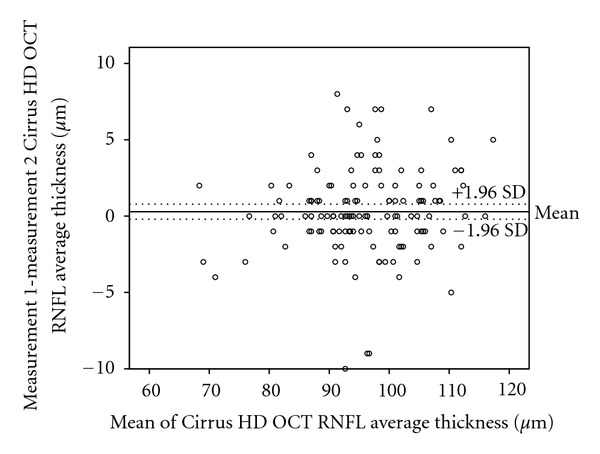
Bland-Altman plots of the agreement in retinal nerve fiber layer (RNFL) thickness between Cirrus HD OCT measurement 1 and Cirrus HD OCT measurement 2. The difference (Cirrus HD OCT average RNFL thickness measurement 1 minus Cirrus HD OCT average RNFL thickness measurement 2) was plotted against the average of the three measurements for the average RNFL thickness Cirrus HD OCT measurements. SD: standard deviation.

**Figure 3:**
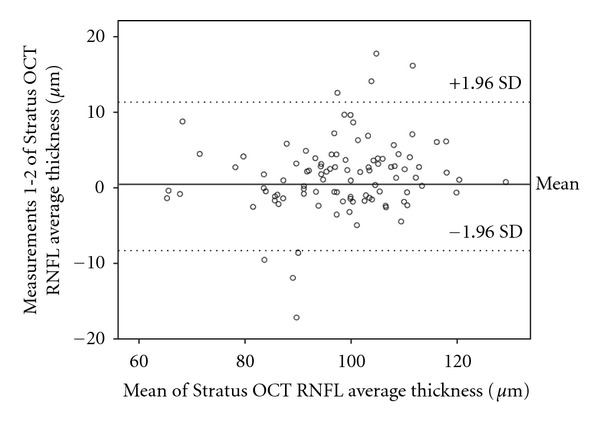
Bland-Altman plots of the agreement in retinal nerve fiber layer (RNFL) thickness between Stratus OCT measurement 1 and measurement 2. The difference (Stratus OCT average RNFL thickness measurement 1 minus Stratus OCT average RNFL thickness measurement 2) was plotted against the average of the three measurements for the average RNFL thickness Stratus OCT measurements. SD: standard deviation.

 Reproducibility is presented in Table 2. Bland-Altman plots were made depicting the agreement between different Cirrus HD OCT RNFL thickness measurements (Figure 2) and Stratus measurements (Figure 3). The difference between Cirrus HD OCT measurements (first Cirrus HD OCT RNFL thickness-second Cirrus HD OCT RNFL thickness) was plotted against the average of the three Cirrus HD OCT measurements for the RNFL thickness (Figure 2). The scatterplots demonstrate the agreement of the measurements. More than 90% of the measurements were between ±5 microns differences (Figures 2 and 3). These small differences were not related to the average RNFL thickness and were similar in those with thinner or smaller thickness. Variability of RNFL measurements was higher with Stratus OCT, as is shown in Tables 1 and 2, and Figure 3.

**Figure 4:**
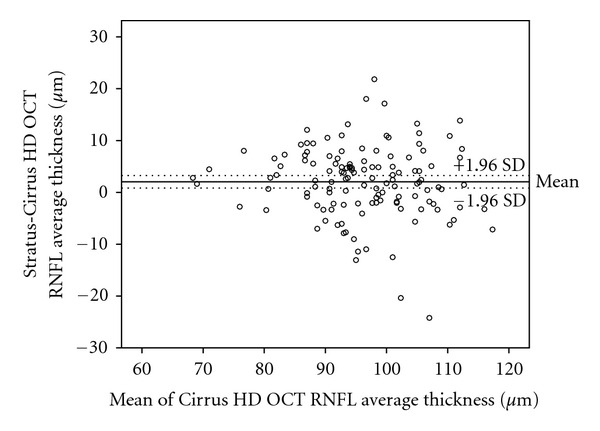
Bland-Altman plots of the agreement in retinal nerve fiber layer (RNFL) thickness between Stratus OCT and Cirrus HD OCT. The difference (Stratus OCT average RNFL thickness minus Cirrus HD OCT average RNFL thickness) was plotted against the average of the three measurements for the average RNFL thickness Cirrus HD OCT measurements. SD: standard deviation.

In most of the cases, direct comparison of RNFL measurements on the same eyes using both OCT instruments showed that the Stratus OCT gave a thinner measurement of the RNFL. As it would be expected, there was high correlation between measurements taken by both devices, and when differences between measurements were evaluated using Bland-Altman plots, considerable discrepancy between the two instruments was observed (Figure 4). Some authors have designed a correction factor to predict the RNFL measurement using other OCT devices, but this analysis is only an approximation. 

As previous authors have described, regional reproducibility data shows the nasal quadrant to be the least reproducible (highest root mean squared error) (Table 2) [18, 22]. When incorporating the RNFL thickness into a reproducibility calculation, the coefficient of variation for the superior and inferior quadrants is smaller than for the nasal and temporal quadrants. This is due to the smaller mean RNFL thickness values in the nasal and temporal quadrants. Gürses-Ozden et al. demonstrated that increasing the number of A scans per acquisition fourfold significantly reduced the coefficient of variation in those quadrants with corresponding visual field defects [26].

RNFL measurements obtained in this reproducibility study are consistent with known properties of the RNFL with thicker superior and inferior nerve fiber bundles in normal eyes. There are many potential explanations for variability in RNFL measurements. Factors such as media opacity, pupil dilatation sampling density, type of scan, and the quadrant measured may all have effects on the overall scan quality and the calculated RNFL thickness [15, 27, 28]. The variability of measurements attributable to different operators and different sessions within the same visit has been shown to be relatively small [28]. In summary, our results indicate that the reproducibility of OCT is adequate for assessing long-term followup for any optic neuropathy or RNFL damage.

## 4. Conclusions

Stratus and Cirrus high definition optical coherence tomography devices give good retinal nerve fiber layer thickness measurements with differences between their values. The reproducibility of RNFL measurements using Stratus time domain OCT is good and excellent with Cirrus HD Fourier domain OCT.

##  Conflict of Interests

The authors have no proprietary interests. No conflicting relationship exists for any author.

## Figures and Tables

**Table 1:** Mean retinal nerve fiber layer parameters and standard deviation measured by Cirrus high definition (HD) and Stratus optical coherence tomography (OCT) and their statistical comparisons.

	Stratus OCT parameters		Cirrus HD OCT parameters		Mean difference (Stratus-Cirrus)	*P*
	Mean	SD	Mean	SD		
Average thickness	97.85	11.47	95.50	9.45	2.37	***0.003***
Superior thickness	119.54	19.47	117.06	17.32	2.63	0.070
Nasal thickness	74.44	19.83	71.41	16.55	2.96	0.106
Inferior thickness	126.38	18.87	125.36	16.63	1.09	0.357
Temporal thickness	70.99	13.62	68.38	11.81	2.21	***0.046***
H1 hour sector	110.65	29.35	103.77	26.41	7.17	***0.004***
H2 hour sector	90.02	26.71	87.49	19.34	2.36	0.317
H3 hour sector	59.84	17.15	56.86	11.99	2.91	0.068
H4 hour sector	73.01	20.62	66.23	16.13	6.83	***0.000***
H5 hour sector	106.28	26.12	101.65	24.34	4.79	***0.006***
H6 hour sector	134.54	27.42	133.72	29.47	0.86	0.657
H7 hour sector	141.86	26.31	138.64	24.48	2.52	0.203
H8 hour sector	75.32	18.56	71.88	18.35	4.17	***0.006***
H9 hour sector	53.40	10.61	53.87	13.77	-0.05	0.968
H10 hour sector	82.24	19.83	82.92	20.52	-0.79	0.620
H11 hour sector	131.51	24.56	126.84	25.65	4.75	***0.023***
H12 hour sector	123.05	25.81	115.65	26.25	7.63	***<0.001***

**Table 2:** Coefficients of variation (COVs) and intraclass coefficients (ICCs) for repeated retinal nerve fiber layer thickness measurements using Cirrus HD and Stratus optical coherence tomography.

	Cirrus HD OCT		Stratus OCT	
	COV	ICC	COV	ICC
Average thickness	1.64	0.985	2.22	0.978
Superior quadrant	3.37	0.970	4.46	0.899
Nasal quadrant	4.80	0.886	4.03	0.950
Inferior quadrant	3.57	0.943	5.78	0.878
Temporal quadrant	3.51	0.956	5.99	0.780
H1	5.30	0.972	6.76	0.956
H2	5.58	0.954	6.08	0.905
H3	4.49	0.957	5.40	0.923
H4	5.33	0.963	5.87	0.938
H5	5.39	0.964	5.94	0.897
H6	5.62	0.947	6.98	0.905
H7	4.24	0.964	5.48	0.938
H8	5.61	0.944	6.91	0.923
H9	3.98	0.931	5.75	0.919
H10	3.80	0.977	4.53	0.969
H11	4.59	0.934	4.96	0.866
H12	6.37	0.936	7.70	0.845

*COVs, coefficients of variation (in %); ICCs, intraclass coefficients.
